# Review of the missed opportunities for the prevention of vertical transmission of HIV in Brazil

**DOI:** 10.6061/clinics/2019/e318

**Published:** 2019-09-10

**Authors:** Mariana Fernandes Guimarães, Kathryn Lynn Lovero, Juliana Gregório de Avelar, Laís Lopes Pires, Giovanna Rodrigues Teixeira de Oliveira, Estela Magalhães Cosme, Camila de Morais Salviato, Thais Raquelly Dourado de Oliveira, Natália Beatriz Cabrera, Claudete Aparecida Araújo Cardoso

**Affiliations:** I Departamento Materno-Infantil, Faculdade de Medicina, Universidade Federal Fluminense, Niteroi, RJ, BR; II Infectious Diseases, Berkeley School of Public Health, University of California, California, US

**Keywords:** Vertical transmission, HIV, Missed Opportunities, Prevention, Children

## Abstract

**OBJECTIVE:**

The present literature review aims to highlight gaps in the treatment of preventative mother-to-child HIV transmission and the risk factors in Brazil.

**METHODS:**

Among the 425 articles identified in SciELO and PubMed searches, 59 articles published between 1994 and 2016 were selected for reading and data extraction, and 33 articles were included in the present review.

**RESULTS:**

The rates of vertical HIV transmission described in the studies varied widely, from 1.8% to 27.8%, with a significant reduction over the years. However, recent rates were also found to be variable in different regions of Brazil, and despite the significant reduction in mother-to-child transmission, many gaps remain in prevention services. A failure to attend prenatal care is the main factor associated with the increased risk of vertical transmission of HIV, hindering early maternal diagnosis and the completion of preventative measures during the prenatal period and, often, the peripartum and postnatal periods. A small number of studies discussed the sociodemographic factors, including a low level of education for pregnant women and the inadequacies of health services, such as difficulties scheduling appointments and undertrained staff, associated with vertical transmission. As such, the current challenge is to better define the sociodemographic and infrastructural factors that increase the risk of mother-to-child transmission of HIV to provide the necessary investments to promote an earlier inclusion of these populations in prevention services.

**CONCLUSIONS:**

This review may serve as a guide for future programs to focus efforts on the prevention of vertical HIV transmission.

## INTRODUCTION

The world has committed to ending the AIDS epidemic by 2030 1. To achieve this goal, increased investments in strategies 1 to prevent new cases of the disease and adequate treatments for individuals already infected with the virus are required 2. Among these strategies, it is critical that substantial efforts be made to reduce vertical transmission of HIV, as this is the main route by which children under 5 become infected 3.

In the absence of adequate prophylaxis, mother-to-child transmission (MTCT) occurs in 20-45% of children born to HIV-infected mothers 4. While the main periods of transmission are gestation and peripartum, approximately 7-22% of cases of vertical transmission occur in the postpartum period through breastfeeding 5,6. Fortunately, in the last 30 years, many advances have been made in the prevention of MTCT. Currently, with the implementation of all the preventative interventions advocated by the National STD and AIDS Program, it is possible to reduce the rates of vertical transmission of HIV to less than 2% 7-9. In addition to the use of antiretrovirals (ARVs), these recommendations include an elective cesarean section for pregnant women with a viral load above 1,000 copies/ml or an unknown viral load, as well as the inhibition of lactation and the utilization of infant formula.

In Brazil, free and universal access to combined antiretroviral therapy (ART) has been offered to pregnant women since 1997. Beginning in 2012, Brazil began offering lifelong triple ART to HIV-infected pregnant women (Option B+), and by 2013, Brazil adopted the Treatment as Prevention (TasP) approach and now offers free ART to all HIV-infected adults. Additionally, since 2002, formula has been provided free of charge during the first six months of a child's life 9. To improve access and promote the use of these prophylactic interventions, the Brazilian Ministry of Health introduced the “Stork Network” (Rede Cegonha) in 2011 to expand and strengthen nationwide MTCT prevention services 10.

Despite advances in preventing MTCT, data have shown that in 2015, 1.8 million children globally were living with HIV, and more than 150,000 were newly infected 1. In Brazil, 7,901 HIV-infected pregnant women were reported in 2015—31.9% in the southeast, 29.6% in the south, 20.9% in the northeast, 11.8% in the north and 5.8% in the central-west 3. In the same year, 62 cases of HIV infection were reported in children under 5 throughout the country 3, and it was estimated that the frequency of MTCT nationally was still between 15 and 24% 11.

This literature review was carried out with the objective of identifying barriers and facilitators to implement prophylaxis and, consequently, to reduce MTCT in Brazil. The study presents the vertical transmission rates of HIV and the missed opportunities in the prevention of MTCT in the different regions of Brazil, aiming to highlight the diversities and the demands of each region, as well as over time; the overall aim was to understand the impact of evolving national policy and services for MTCT over the years.

## METHODS

Articles were identified in PubMed using the following search terms: “Risk Factors Vertical Transmission Brazil”, “Risk Factors Mother-to-Child Transmission”, “Vertical Transmission HIV Brazil” and “Mother-to-Child Transmission HIV Brazil.” We also searched for articles in SciELO using the following terms: “Transmissão vertical HIV Brasil”, “Fatores de risco transmissão vertical Brasil”, “Transmissão materno infantil HIV Brasil” and “Fatores de risco transmissão materno infantil Brasil.” Articles published between 1994 and 2016 in English and Portuguese were considered for inclusion in the review.

**Figure 1:**
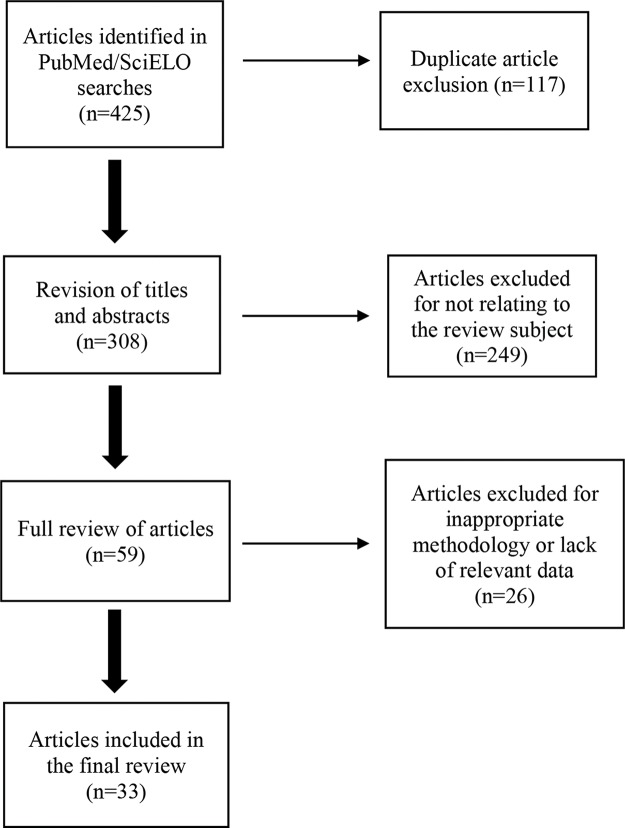
Flowchart of the selection of articles included in the literature review.

Among the 425 search results, 59 articles were selected (according to the title and abstract) for reading and extracting data. The exclusion criteria were an inappropriate methodology and articles that did not present information on rates and/or factors directly associated with MTCT. A total of 33 analytical and descriptive studies were included, from which it was possible to obtain information on the rates of vertical HIV transmission in the different regions of Brazil, the main factors associated with the increase in these rates, and the difficulties faced in providing prophylaxis. Figure 1 - Flowchart of the selection of articles included in the literature review - shows the selection process of the articles. In addition, classic literature articles on the topic and protocols were also read and are cited in the present review.

For presentation of the results of our review, missed opportunities and the risk factors associated with MTCT were grouped by perinatal period as follows: prenatal care, peripartum care, and postnatal care. Sociodemographic risk factors and inadequacies in MTCT delivery were also evaluated.

## RESULTS

### Rates of HIV transmission by geographical region

A total of 33 articles were selected based on an evaluation of the methodology and the results of interest. Among the included studies, 24 had information on the rates of vertical transmission of HIV according to the region of the country. Table 1 highlights these data.

The rates of MTCT in Brazil varied from 1.8% in a study by Lemos et al. 14 in Sergipe to 27.8% in a study by Turchi et al. 30 in Goiânia. This divergence may be explained by the period of the studies, the former being conducted between 2010 and 2011, and the latter conducted between 1995 and 2001. Supporting this, it was possible to observe a substantial reduction in MTCT rates over the years (Table 2). In a southeast capital of the country, for example, there was an 85% reduction in the MTCT rates between 1998 and 2005 24.

### Factors associated with increased vertical transmission rates

#### 1) Prenatal care

Diagnosis of maternal HIV infection during the prenatal period is critical to applying complete preventative care for MTCT. In Brazil, following the protocol of the Ministry of Health, HIV testing of the mother by ELISA or a rapid test should be carried out in the first trimester and, in the case of a negative first test, should be repeated in the third trimester. If the mother is infected with HIV, she should be counseled about preventative treatments and receive joint monitoring in a basic health unit and in an HIV reference unit 9.

In different regions of the country, failure to attend prenatal care 13,17,37, late maternal diagnosis 13,37, and an incomplete ART regimen during pregnancy 13,16,17,37 were highlighted as significant risk factors for MTCT. A retrospective study conducted between 2000 and 2009 in the northeast of the country in the state of Pernambuco demonstrated the importance of prenatal care in the prophylaxis of vertical HIV transmission. Pregnant women who underwent prenatal care had only an 8.1% (68/845) MTCT rate, whereas in the group that did not perform prenatal care, this number increased to 27.1% (19/70) 16. It is important to note that 7.7% (70/915) of the pregnant women remained without prenatal care in this study.

Concerning data on prenatal care attendance was shown in this same region through a cross-sectional study carried out at a public maternity hospital in Salvador in 2001, in which 29.6% of the women stated that they had not attended any prenatal care 38. Similarly, inadequate rates of prenatal attendance were also observed in studies conducted in the southeast region. Nishimoto et al. 19 showed that in a cohort study from 1997 to 2000 in the state of São Paulo, 20.4% (29/142) of the HIV-infected pregnant women participating in the study did not attend prenatal care. More recently, in Rio de Janeiro, a sample of 40 HIV-infected participants born in 2008 who had not received HIV transmission prophylaxis was evaluated. Of these women, 30% did not attend prenatal care, 50% had 1 to 5 consultations, and 20% had 6 or more consultations 39. Another study in Rio de Janeiro, evaluating a cohort of HIV-infected pregnant women between 1997 and 2014, found that 14% of these women did not attend any prenatal care 40. Two studies we identified evaluated prenatal care attendance over the years and observed a modest increase in attendance rates. A study of 1364 HIV-infected pregnant women between 2000 and 2009 across the northeast state of Ceará found an increase in prenatal attendance over the study period, although just 84.5% of women had attended any prenatal care in the final study year 37. In the Rio de Janeiro cohort of HIV-infected pregnant women from 1997 to 2014, prenatal care attendance increased over the study period, although again just 83.3% of HIV-infected pregnant women had at least one prenatal visit in 2014 40.

The period of maternal HIV diagnosis also showed a direct association with the rates of MTCT. A study carried out from 1995-2001 in Goiânia 30 showed that the risk of transmission was significantly higher in women diagnosed postpartum (40.8%, 62/152) than in women diagnosed prepartum (4.8%, 8/166). In the northeast region, Cruz Gouveia et al. 16 demonstrated that, of the pregnancies with a maternal diagnosis made before or during gestation between 2000 and 2009, the rate of infected children was 4.7%; when the diagnosis was made during or after delivery, this rate increased to 25.8%. In the southeast region, Lovero et al. 40 found that, in a cohort of HIV-infected pregnant women from Rio de Janeiro between 1997 and 2014, a postpartum diagnosis was associated with an eleven-fold increase in the likelihood of MTCT.

Across the different regions of Brazil, the rates of prenatal diagnosis varied considerably by region. Two retrospective studies conducted in the central-western and southeast regions of the country between 1996-2001 and 1999-2004, respectively, showed that most HIV-infected pregnant women knew about the diagnosis before or during prenatal care, but there were still women diagnosed only at the time of delivery 29,21. In the first study, 51.3% (39/76) of pregnant women learned about their HIV status during the prenatal period, while 40.8% (31/76) had the diagnosis prior to gestation. The remaining six women (7.9%) learned of the diagnosis at the time of delivery through HIV rapid testing performed during delivery. In the second study, in a sample of 44 pregnant women, 47.7% were aware of their diagnosis before gestation, 50% during gestation and 2.3% at the time of delivery. More concerning data were demonstrated in a study by Nishimoto et al. 20 conducted from 1997 to 2000 in the southeast region, in which only 51.8% of the HIV-infected pregnant women were tested for HIV infection during prenatal care.

More recently, in a study conducted in the northern region of Brazil between 1999 and 2011, 32.3% (297/919) of pregnant women already knew their HIV diagnosis before pregnancy, 48.9% (449/919) were diagnosed during prenatal care, 7.3% (67/919) were diagnosed during the intrapartum period, and 11.5% (106/919) only had this diagnosis in the postpartum period, missing any opportunity for prophylactic treatment 13. Similar results were identified in a study of HIV-infected pregnant women from Rio de Janeiro between 1997 and 2014, in which 11.5% (50/435) did not receive an HIV diagnosis until after delivery. Additionally, while this study found an increase in prenatal HIV diagnosis across the study period, just 83% of mothers were diagnosed before or during the prenatal period in 2014 40.

Following an evolution in national policy and an increase in the availability of antiretrovirals over the last 20 years, the use of ART during pregnancy has substantially increased across many regions of Brazil. In the southeast region, the use of combined ART increased from 0% in the period prior to nationwide availability between 1990 and 1994 to 46.4% between 1999 and 2000 23. Kakehasi et al. 24 also observed an increase in the use of prenatal ART over time in the southeast region, from 66.7% in 1998 to 89.9% in 2005. A study conducted in the south of the country, in the State of Rio Grande do Sul, showed 60.7% prenatal ART adherence from 1998-2004 and 73.3% from 2005-2011 35. Additionally, in the northern region, the number of women receiving prenatal ARVs increased from 70.8% in 1999-2000 to 79.4% in 2011 13. In Rio de Janeiro, the use of prenatal ART also increased between 1997 and 2014, although 16.7% of HIV-infected pregnant women did not use ART in 2014 40.

In the northeast region, Cruz Gouveia et al. 16 emphasized an increased risk of MTCT when pregnant women started the medication in the third trimester of pregnancy. However, there is a lack of information in the literature about the gestational age when ART was initiated in other regions of the country.

#### 2) Peripartum measures

In addition to the missed opportunities in prenatal care, measures to prevent MTCT during childbirth remain a challenge in Brazil. In the intrapartum period, prevention measures are based on the method of delivery (cesarean section is recommended for women with a viral load >1,000 copies/ml or an unknown viral load), the time of ruptured membrane (ideally the rupture occurs in the delivery room), the HIV rapid test (done for all pregnant women who did not attend prenatal care or have unknown/uncertain HIV status), and the administration of azidothymidine (AZT) in the delivery room, both to the mother and the baby 41.

Several studies have shown that elective cesarean section is a protective factor for vertical transmission of HIV in Brazil 13,16,37. Between 2006 and 2007 in the city of São Paulo, vaginal delivery was chosen by 13.3% (6/45) of the pregnant women who were using ART with an undetectable viral load. Cesarean section was performed for 86.7% (39/45) of pregnant women—71.1% (32/45) elective, 13.3% (6/45) emergency, and 2.2% (1/45) without prior knowledge 42. In a retrospective study in the northern region between 1999 and 2011, 20.9% (200/959) of women delivered vaginally, 19.8% (190/959) received an emergency cesarean section, and 59.3% (569/959) received an elective cesarean section. For the women who delivered by an elective cesarean section, the rate of MTCT was 2.5%, while in the group that did not, this number increased to 10.8% 13. A study by Kakehasi et al. 24 was conducted from 1998-2005 in the capital of the southeast region and showed a variation in the rates of elective cesarean sections over the years, with an increase up to 2000, followed by a downward trend in the following years of the study. In 1998, 40% of deliveries were elective cesarean sections. This number practically doubled by 2000 and then decreased again, reaching 54.2% in 2005.

The use of intravenous AZT during delivery was also directly associated with a reduction in the rates of MTCT 13,14,17,37. In a study by Cruz Gouveia et al. 16 in the northeast, 75% of pregnant women received intrapartum AZT between 2000 and 2009, and the rate of MTCT decreased from 21.8% to 4.6% in women who received AZT during delivery. In the north of the country, 80.6% of pregnant women between 1999 and 2011 received intrapartum AZT 13. The rate of MTCT for women who had not used intrapartum AZT was 19.6%, whereas the rate of MTCT was just 3.3% for those who had 13. In the southeast of the country, 53.1% of women received AZT during delivery between 1997 and 2014, and the absence of intrapartum AZT was associated with a 4.9-fold increase in the likelihood of MTCT 40. A study by Lima et al. 37 in the northeast region demonstrated that ARV use at the time of delivery increased between 2000 and 2009, with 67% of the women receiving AZT during delivery in 2009.

In addition, Cruz Gouveia et al. 16 emphasized premature birth (gestational age less than 37 weeks) as a risk factor for MTCT. Out of 113 children born at less than 37 weeks, 16.8% were infected with HIV, while of those with gestational ages greater than or equal to 37 weeks, only 8.7% were infected. A study in the southern region of the country also showed a significant association between membrane rupture time and the reduction in vertical HIV transmission rates over the years. From 1998-2004, membrane rupture time was greater than 4 hours in 79.4% of the pregnant women evaluated; in the period between 2005 and 2011, only 10.8% of women had membrane rupture times greater than 4 hours 35.

#### 3) Postnatal care

In the postpartum phase, follow-up is needed for both the mother, with continued HIV control and care to ensure that she is not breastfeeding, as well as for the newborn, who is required to receive oral AZT for four weeks if the mother completed the prenatal prophylaxis. If prenatal prophylaxis was not completed and/or the maternal viral load is unknown or up to 1,000 copies/ml at delivery, since 2014, 3 doses of nevirapine have been added to this regimen 43,44. The child's HIV viral load should be measured at four weeks of age and after four months. If both have undetectable results, the child can be declared HIV negative. Children exposed vertically to HIV should continue to receive formula up to 6 months of age to avoid MTCT via breastfeeding 42. Studies in Brazil have shown that missed opportunities for the prevention of MTCT during the postpartum period include breastfeeding 13,16,24, maternal diagnosis of HIV after childbirth, and lack of ART for the newborn 14,37,45.

A study was conducted by Kakehasi et al. 24 in the southeast of the country and found that breastfeeding was significantly more common in children infected with MTCT, with 33.9% of HIV-infected infants being breastfed compared to just 4.3% of uninfected children. The Brazilian Ministry of Health protocol recommends against breastfeeding, and free formula has been supplied for HIV-exposed infants since 2002; a study by Andrade et al. 13 evaluated births between 1999 and 2011 in the north of the country and another study evaluated births between 1997 and 2014 in the southeast city of Rio de Janeiro 40, and these studies found that approximately 10% of HIV-exposed children were breastfed. Both studies demonstrated that this was largely due to the late diagnosis of maternal HIV. Additionally, Cavalcante et al. 45 showed that among 10 women who did not have access to prenatal care between 2002 and 2003 in the northeast city of Fortaleza, only one of their infants was not breastfed. In the study by Andrade et al. 13, a ten-fold decrease in breastfeeding was observed between 1999 and 2011, although 3.2% of babies exposed to HIV were still breastfeed in 2011.

#### 4) Sociodemographic factors

In addition to the biological factors associated with an increased risk of MTCT, it is important to address several social factors that directly or indirectly hinder pregnant women's access to MTCT prevention services 28.

In the northeast and central-west region of the country, the low education level of pregnant women was highlighted as a possible limiting factor for the full implementation of preventative measures 14,30,37. Between 1994 and 2010, a study carried out in the state of Sergipe in the northeast region showed that 58.2% of HIV-infected pregnant women had less than seven years of formal education completed 14. It was similar in Goiânia between 1995 and 2001, where it was shown that 82.4% of HIV-infected pregnant women had less than eight years of schooling 30. In a descriptive study conducted in Belo Horizonte between 2004 and 2005, Lana et al. 46 concluded that women with less education who depend on public health services are less likely to initiate prenatal care early in pregnancy or to complete the recommended number of prenatal visits.

Among studies assessing the sociodemographics of HIV-infected pregnant women, there was a great deal of homogeneity among family income, with the majority of women coming from a household of one to two and a half times the monthly minimum wage. Nishimoto et al. 20 found an average income of 2.5 times the minimum wage, with 29% of women making a maximum of one minimum wage salary per capita per month. According to Araujo et al. 28, despite the significant decrease in poverty in Brazil in recent years, the country remains with serious social, economic and cultural inequities. These inequalities include disparities related to the health system, and these disparities are evident regarding access to prenatal care and greatly impede the success of programs to prevent vertical transmission of HIV 28.

Finally, an important finding that serves as motivation for the performance in the area of education appears in a study by Fernandes et al. 21 in the southeast region: most pregnant women (70.5%) are aware of the possibility of transmission of HIV to their child. If this trend continues, it is hopeful that the rate of prenatal adherence will increase and, in turn, the rate of MTCT in Brazil will decrease.

#### 5) Inadequacies in health services

In addition to the sociodemographic characteristics of HIV-infected mothers, inadequacies in preventative care for MTCT can contribute to increased MTCT rates. A study was carried out between 2004 and 2005 in a basic health unit in the northeast region in the State of Ceará; this study highlighted that the deficit in the recruitment of pregnant women, the delay in scheduling prenatal visits by the community agent, and the delay in completing the HIV test, and all constituted important institutional barriers to MTCT prevention 47.

It is also worth noting the enormous importance of making pregnant women feel welcome at health centers to promote prenatal care uptake and adherence and to avoid the first step in a cascade of missed opportunities. In a study by Darmont et al. 39, interviews with 40 pregnant women from Rio de Janeiro in 2008 showed that many HIV-infected women abandon care because of issues in healthcare delivery, such as excessive bureaucracy to make an appointment, the high turnover of health professionals at health posts, and no prioritization of care for pregnant women.

Additionally, a study by Farias et al. 48 evaluated the knowledge of MTCT prevention measures in obstetricians at public maternity hospitals in Salvador in 2005 and found that only 82% reported a full understanding of the Ministry of Health's recommendations. The factors that prevented the completion of preventative measures were assessed, and 74.4% of obstetricians reported inadequate prenatal follow-up visits, 50% said it was due to a lack of information obtained during prenatal care at the time of maternity admission, 42.6% reported work overload, and 9% cited an inadequate disclosure of recommendations. The lack of training programs was also raised by 38.8% of these professionals since only 41.9% of those interviewed attended a course or internship for HIV training.

## DISCUSSION

In the present review, we found that the reported rates of MTCT varied considerably across different regions of Brazil, ranging from 1.8% in a cohort from the northeast region to 28.8% in a cohort from the central-west region. Further evaluation indicated that these regional differences were largely due to the period in which the study was conducted, as studies directly comparing MTCT rates over time demonstrated a decrease in cases over the years, similar to global reductions in MTCT rates 49. However, a multicenter study carried out in Brazil between 2008 and 2009 indicated that the interregional differences in MTCT rates also occurred even within the same study period, with the north and northeast regions having much higher rates of MTCT (8-18%) than the south and central-west regions (4-7%) 50. Moreover, disparities were apparent even within the same region, with higher rates of MTCT in metropolitan regions 25,28.

Assessing risk factors for the prevention of MTCT, we identified missed opportunities across regions in the prenatal, peripartum, and postnatal periods. Although reductions in these missed opportunities were observed over time, global goals for the elimination of MTCT 51 are still not being met.

For the MTCT prevention routine to be effective, early diagnosis is essential. Easy access to and robust services in prenatal care remain the main pillars in the reduction in MTCT. We found that attendance in prenatal care across regions ranged from ∼70-90%. Two studies were conducted, one in the southeast city of Rio de Janeiro 40 and one in the northeast state of Ceará 37, and both demonstrated an increase in prenatal care engagement over time. In 2011, the Stork Network (“Rede Cegonha”) was introduced to expand and improve MTCT prevention services across Brazil. The one study we identified that presented prenatal attendance data after the creation of the Stork Network 40 found an improvement in prenatal attendance from 2011-2014, but in 2014, only 83% of HIV-infected pregnant women had attended at least one prenatal visit. Additional recently conducted research is needed to determine whether the improvement in prenatal attendance following the creation of the Stork Network has continued and whether the global goals of 95% engagement in prenatal care 51 will be met in Brazil.

Despite the recent global increase in the number of pregnant women using ARVs, it is still estimated that more than 20% of pregnant women in the world are not receiving these medications to prevent MTCT 49. In addition, in the same year in Latin America and the Caribbean, the percentage of pregnant women with HIV receiving some ARVs during pregnancy was just 54% and 52%, respectively 8. In Brazil, we found substantial improvements in the use of prenatal ART in the years following the introduction of universal, free access to HAART in 1997. Moreover, one study 40 evaluated MTCT prevention after the 2012 adoption of Option B+ (lifelong triple ART to HIV-infected pregnant women) and showed continued increases in prenatal ART use. Nevertheless, in 2014, prenatal ART coverage was 83%, below the global elimination target of 90% 51. Similar to engagement in prenatal care, it will be important for ongoing studies to determine if the use of prenatal ART has continued to increase in other regions of Brazil and in more recent years or if additional efforts are needed to reach global goals.

With regard to preventative services in the peripartum period, we found that cesarean sections have decreased over the years in Brazil 24. While these low rates of cesarean sections may reflect a missed opportunity for the prevention of MTCT, these data may also reflect the increased use of prenatal ART and, in turn, an increased control of maternal viral loads during the peripartum period over the years. Further research will need to be carried out to determine the percentage of women requiring but not receiving cesarean sections for MTCT prevention throughout Brazil. In the postnatal period, missed opportunities, including breastfeeding 13,16,24,40 and a lack of ART for the newborn 14,37,45, were observed and were most often due to the maternal diagnosis not occurring until the postnatal period. Together, these findings on missed opportunities in the peripartum and postnatal periods highlight the importance of prenatal care and early maternal diagnosis in ensuring that all prophylactic measures are performed.

Among the articles analyzed, very few presented information about sociodemographic factors related to the prevention of MTCT. In future studies, sociodemographic factors associated with the increased risk of MTCT, especially factors that make it difficult for pregnant women to access or continue prenatal care, will need to be determined to guide new public health measures to target vulnerable populations.

An important limitation of the studies reviewed is that most lacked information on the week of gestation in which ART was initiated, which is fundamental to the analysis of uptake and the quality of prenatal care offered. Additionally, the analysis of the data presented here should be done with caution since the data mainly reflect information from large cities and reference centers and may not represent the reality of the entirety of Brazil. For example, in the northern region, the two articles analyzed were carried out in Manaus, and we did not find information about other cities. For a more complete understanding of missed opportunities in the prevention and risk factors for MTCT nationwide, new studies should be directed to less explored regions.

Our review of studies throughout Brazil shows a substantial drop in the rates of MTCT and an increase in adherence to prophylactic measures over the years. Despite this, there are still many pregnant women who do not benefit from all the recommended interventions for HIV prevention 32. To achieve the global elimination of MTCT goal 51, it is essential to improve and facilitate the population's access to prenatal care. Through these measures, it will be possible to provide an early diagnosis and promote the completion of all MTCT preventative measures.

## AUTHOR CONTRIBUTIONS

Guimarães MF and Lavero KL selected the articles to read. Guimarães MF, Avelar JG, Pires LL, Oliveira GRT, Cosme EM, Salviato CM, Oliveira TRD and Cabrera NB read the articles, organized and interpreted the data, and wrote the manuscript. Guimarães MF, Avelar JG, Pires LL, Oliveira GRT and Cosme EM formatted the version to be published. Lavero KL and Cardoso CAA contributed to the conception and relevant critical revision of the intellectual content. All authors contributed to the final approval of the manuscript to be published.

## Figures and Tables

**Table 1:** Rates of vertical transmission of HIV in different regions of Brazil.

Region	City	Article	Study Population	Study Design	Study Period	Rate of Vertical Transmission
North	Manaus - AM	Soeiro et al. 2011 (12)	509 HIV-infected pregnant women. Data provided by the Coordenação Estadual do Amazonas para Doenças Sexualmente Transmissíveis / Aids.	Descriptive study	2007 - 2009	9.9%
	Manaus - AM	Andrade et al. 2015 (13)	1037 HIV-exposed infants admitted before 18 months of age at the pediatric HIV reference service of the Fundação de Medicina Tropical Doutor Heitor Vieira Dourado (FMT-HVD).	Retrospective cohort study	1999 - 2011	6.6%
Northeast	Aracaju - SE	Lemos et al. 2012 (14)	110 HIV-infected pregnant women and their infants admitted to the Hospital Maternidade Nossa Senhora de Lourdes.	Retrospective descriptive study	1994 - 2010	1.8%
	Sergipe	Lemos et al. 2013 (15)	561 HIV-exposed infants in Sergipe. Data were obtained through the Sistema de Informações de Agravos de Notificações (SINAN), the Sistema de Informações sobre Mortalidade (SIM), the state laboratory database, and the medical records at the Universidade de Sergipe and CEMAR (Centro de Especialidades Médicas para HIV / DST / AIDS).	Retrospective cohort study	1990 - 2011	18.9%
						
	Recife - PE	Gouveia et al. 2013 (16)	1015 HIV-exposed infants born in Pernambuco and registered by 2 months of age in the public program for prevention of vertical transmission (IMIP).	Retrospective cohort study	2000 - 2009	9.2%
	Salvador - BA	Patricio et al. 2015 (17)	622 HIV-exposed children 0-36 months of age born in Bahia to mothers treated at the Centro de Referência de Diagnóstico e Pesquisa de Doenças Sexualmente Transmissíveis e HIV / AIDS do Estado da Bahia (CEDAP).	Cross-sectional study	2005 - 2008	8.2%
						
	Petrolina - PE e Juazeiro - BA	Brandão et al. 2016 (18)	76 mother-child pairs treated in the cities of Petrolina and Juazeiro.	Cross-sectional study	2006 - 2010	8.6%
Southeast	Santos, Ribeirão Preto, São Paulo e Campinas - SP	Tess et al. 1998 (19)	434 HIV-exposed children born in 4 different hospitals in cities throughout the state of São Paulo.	Retrospective cohort study	1988 - 1993	16%
	Santos - SP	Nishimoto et al. 2005 (20)	144 HIV-infected mothers and their children recruited from the HIV reference center, 19 basic health units, and 2 maternity centers in the city of Santos.	Retrospective cohort study	1997 - 2000	9.7%
	Campos dos Goytacazes - RJ	Fernandes et al. 2005 (21)	44 HIV-infected pregnant women and their children treated at the HIV/AIDS program of Campos dos Goytacazes.	Retrospective descriptive study	1999 - 2004	6.8%
	Vitória - ES	Miranda et al. 2006 (22)	208 HIV-infected pregnant women selected from the Sistema Nacional de Vigilância do Ministério da Saúde.	Retrospective descriptive study	1997 - 2001	3.1%
	Campinas - SP	Amaral et al. 2007 (23)	197 HIV-infected pregnant women and their children, treated at the Centro de Atenção Integral à Saúde da Mulher (CAISM), part of the Universidade Estadual de Campinas (UNICAMP).	Retrospective cohort study	1990 - 2000	11.9%
	Belo Horizonte - MG	Kakehasi et al. 2008 (24)	900 HIV-infected pregnant women and their children admitted at up to 3 months of age to the Centro de Referência em Doenças Infecciosas e Parasitárias Orestes Diniz (UFMG/PBH).	Prospective cohort study	1998 - 2005	6.2%
	Rio de Janeiro - RJ	Veloso et al. 2010 (25)	3778 pregnant women with unknown serological status at delivery in high and mild complexity maternity centers of a metropolitan area.	Cross-sectional study	2000 - 2002	14.6%
	Vitória - ES	Vieira et al. 2011 (26)	137 HIV-infected pregnant women and 14 children vertically infected with HIV, according to records from the Sistema de Informação de Agravos de Notificação (SINAN).	Ecological study	2000 - 2006	9.7%
	Presidente Prudente - SP	Prestes-Carneiro et al. 2012 (27)	86 HIV-infected pregnant women and their children treated at the Hospital Estadual de Presidente Prudente.	Retrospective cohort study	2002 - 2007	4.6%
	Nova Iguaçu - RJ	Araujo et al. 2014 (28)	997 HIV-infected pregnant women and 1259 newborns exposed to HIV treated at the Hospital Geral de Nova Iguaçu (HGNI).	Retrospective cohort study	1999 - 2009	4.7%
Central-West	Campo Grande - MS	Dal Fabbro et al. 2005 (29)	76 HIV-infected pregnant women and their children that were treated at the reference department for HIV-infected women.	Prospective cohort study	1996 - 2001	2.5%
	Goiânia - GO	Turchi et al. 2007 (30)	276 HIV-infected pregnant women and their children that were followed in the public health service.	Hybrid cohort study	1995 - 2001	27.8%
South	Rio Grande - RS	Martínez et al. 2005 (31)	102 HIV-exposed children treated at the AIDS service of the Hospital Universitário Dr. Miguel Riet Correa Jr. (FURG).	Cross-sectional study	1998 - 2004	11.8%
	Porto Alegre - RS	Veloso et al. 2010 (25)	1439 pregnant women with unknown serological status at delivery admitted to high and mild complexity maternity centers in a metropolitan area.	Cross-sectional study	2000 - 2002	11.7%
	Rio Grande - RS	Tornatore et al. 2010 (32)	144 HIV-exposed children treated at the Hospital Universitário Dr. Miguel Riet Correa Jr. (FURG).	Retrospective cohort study	2003 - 2007	4.9%
	Itajaí - SC	Kupek et al. 2012 (33)	15,098 pregnant women and 243 HIV-exposed newborns. Data collected through state and city records.	Retrospective longitudinal observational study	2002 - 2007	6.3%
	Santa Maria - RS	Hoffmann et al. 2016 (34)	198 HIV-infected pregnant women and their children born at the Hospital Universitário de Santa Maria.	Cross-sectional study	2008 - 2012	2.4%

**Table 2:** Reduction in the rates of vertical transmission over the years in different regions of Brazil.

Region	City	Article	Study Population	Study Design	Variation in Transmission Rates over Time
North	Manaus - AM	Andrade et al. 2015 (13)	1037 HIV-exposed infants admitted before 18 months of age at the pediatric HIV reference service of the Fundação de Medicina Tropical Doutor Heitor Vieira Dourado (FMT-HVD).	Retrospective cohort study	7.5% (2007-2008) and 3.2% (2011)
Southeast	Santos - SP	Nishimoto et al. 2005 (20)	144 HIV-infected mothers and their children recruited from the HIV reference center, 19 basic health units, and 2 maternity centers in the city of Santos.	Retrospective cohort study	19.6% (1997); 7% (1998); 3.6% (1999)
	Campinas - SP	Amaral et al. 2007 (23)	197 HIV-infected pregnant women and their children, treated at the Centro de Atenção Integral à Saúde da Mulher (CAISM), part of the Universidade Estadual de Campinas (UNICAMP).	Retrospective cohort study	32.3% (1990-1994); 25.7% (1995-1996); 2.2% (1997-1998) and 2.9% (1999-2000)
	Belo Horizonte - MG	Kakehasi et al. 2008 (24)	900 HIV-infected pregnant women and their children admitted at up to 3 months of age to the Centro de Referência em Doenças Infecciosas e Parasitárias Orestes Diniz (UFMG/PBH).	Prospective cohort study	20% (1998) and 3% (2005)
South	Rio Grande do Sul	Rosa et al. 2014 (35)	353 HIV-exposed infants treated at the HIV reference service of the Hospital Universitário do Rio Grande do Sul (HU-FURG).	Descriptive retrospective study	11.8% (1998-2004) and 3.2% (2005-2011)
Multiregional	North—Amazonas, Pará e Amapá; Região Northeast – Alagoas, Bahia, Ceará, Maranhão, Paraíba, Pernambuco e Sergipe; Central-West – Goiás, Mato Grosso e Distrito Federal; Southeast—Espírito Santo, Minas Gerais, Rio de Janeiro e São Paulo; South—Rio Grande do Sul, Paraná e Santa Catarina	Succi et al. 2007 (36)	2924 HIV-exposed infants from 63 health centers located in 5 regions of Brazil.	Cross-sectional study	8.6% (2000) and 7.1% (2001)
